# Respiratory issues in patients with multiple sclerosis as a risk factor during SARS-CoV-2 infection: a potential role for exercise

**DOI:** 10.1007/s11010-022-04610-1

**Published:** 2022-11-21

**Authors:** Omid Razi, Ana Maria Teixeira, Bakhtyar Tartibian, Nastaran Zamani, Beat Knechtle

**Affiliations:** 1 Department of Exercise Physiology, Faculty of Physical Education and Sport Sciences, Razi University, Kermanshah, Iran; 2 Research Center for Sport and Physical Activity, Faculty of Sport Sciences and Physical Education, University of Coimbra, Coimbra, Portugal; 3 Department of Exercise Physiology, Faculty of Physical Education and Sports Sciences, Allameh Tabataba’i University, Tehran, Iran; 4 Department of Biology, Faculty of Science, Payame-Noor University, Tehran, Iran; 5 Institute of Primary Care, University of Zurich, Zurich, Switzerland; 6 Medbase St. Gallen Am Vadianplatz, Vadianstrasse 26, 9001 St. Gallen, Switzerland

**Keywords:** Multiple sclerosis, COVID-19, Exercise training, Renin–angiotensin system, Respiratory system, Immune system

## Abstract

Coronavirus disease-2019 (COVID-19) is associated with cytokine storm and is characterized by acute respiratory distress syndrome (ARDS) and pneumonia problems. The respiratory system is a place of inappropriate activation of the immune system in people with multiple sclerosis (MS), and this may cause damage to the lung and worsen both MS and infections.

The concerns for patients with multiple sclerosis are because of an enhance risk of infection with severe acute respiratory syndrome coronavirus 2 (SARS-CoV-2). The MS patients pose challenges in this pandemic situation, because of the regulatory defect of autoreactivity of the immune system and neurological and respiratory tract symptoms. In this review, we first indicate respiratory issues associated with both diseases. Then, the main mechanisms inducing lung damages and also impairing the respiratory muscles in individuals with both diseases is discussed. At the end, the leading role of physical exercise on mitigating respiratory issues inducing mechanisms is meticulously evaluated.

## Introduction

The respiratory system, anatomically and functionally, is designed to provide and eliminate oxygen and carbon dioxide (CO_2_), respectively, or simply viewed as a gas exchange system [1, 2]. To do so, the respiratory cycle consists of inspiration and expiration which are performed by the help of several muscles. All components of respiratory system, such as pleurae, airway, and vessels, are innervated by afferent and efferent of autonomic nervous system, sympathetic, and parasympathetic nerves especially the vagal nerve. Breath is an autonomic and rhythmic action that is produced by networks of neurons originating from the brainstem, known as pons and medulla oblongata. These neuronal networks enervate thoracic and abdominal muscles. Three main neuronal groups are involved in monitoring the breath rhythm and its duration: (1) inspiratory neurons in dorsomedial medulla, (2) inspiratory and expiratory neurons in ventrolateral medulla, and (3) inspiratory and expiratory discharging neurons in rostral pons. The important characteristic of this system is its ability to modulate breathing patterns in response to changing of external and internal environments [1, 3, 4].

The majority of components of the respiratory system are impaired in some neurological diseases, such as multiple sclerosis (MS) and Alzheimer disease (AD) [5, 6], and this condition may impose further endangering of these individuals during respiratory virus diseases, like the worldwide coronavirus disease-2019 (COVID-19) pandemic. In this context, physicians most often solicit the use of inhaled steroids and also antibiotic medications [7]. Respiratory pathogenesis of both COVID-19 and MS is extensively referred for improper activation of the immune system, renin–angiotensin system (RAS) dysfunction, the existence of some plaques in brain areas monitoring ventilation skeletal muscles [8–13]. Exercise training as a non-pharmacological intervention by several mechanisms, such as improving the immune responses, converting negative RAS axis to positive one, alleviating the plaque progression, can largely mitigate respiratory issues [14]. Thus, the purposes of this narrative review are to meticulously investigate respiratory issues associated with COVID-19 and MS diseases and also better understand the cellular and molecular mechanisms by which neuro-inflammatory autoimmune disease influences lung immunity. Finally, shed light on the positive roles of regular exercise training as a prophylactic or modifying intervention in mitigating such problems is another outstanding aim of this study.

### Respiratory dysfunctions common road between coronavirus and multiple sclerosis

Severe acute respiratory syndrome coronavirus 2 (SARS-CoV-2) is the third coronavirus disease originated from animal and it belongs to beta-coronaviruses which induce a disease known as novel COVID-19 [15, 16]. As inferred naturally from the name of the virus, this disease is associated with respiratory infections [17].

In most cases, the disease is without any respiratory signs; however, all sufferers may later manifest different degrees of lung disorders due to damages in lung tissue [18].

Acute respiratory distress syndrome (ARDS) and pneumonia are the common clinical manifests in patients with severe COVID-19 [18–21]. ARDS is clinical disorder associated with systemic inflammation and failure in multiple organs with a high mortality rate related to lung damage [22, 23]. Hence, COVID-19 is associated with some disorders in lung tissue, including airways, lung parenchyma, lung vessels, and neuromuscular disruptions [24].

**Fig. 1:**
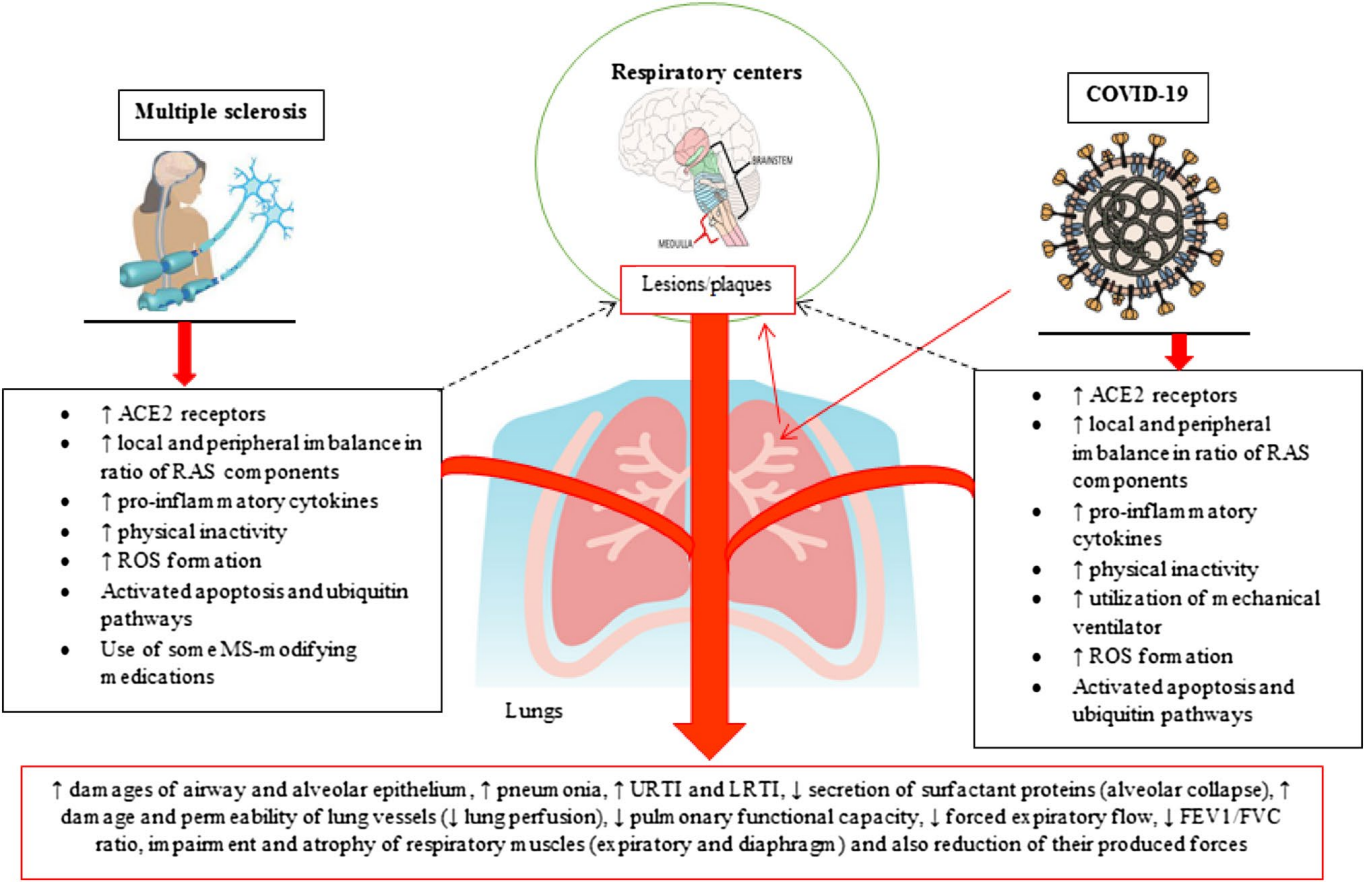
The main pathways inducing structural and functional pathogenesis of lung tissue through both MS and COVID-19 diseases. The red arrows represent the detrimental events that result from both diseases causing the whole pulmonary issues indicated in the red rectangular box below. *ACE2* angiotensin-converting enzyme 2; *RAS* renin–angiotensin system; *ROS* reactive oxygen species; *MS* multiple sclerosis; *URTI* upper respiratory tract infection; *LRTI* lower respiratory tract infection; FEV1/FVC ratio, forced expiratory volume in 1 s to forced vital capacity ration

This virus can infect several systems, including digestive, genitourinary, central nervous system (CNS), and respiratory systems [16, 25, 26]. During COVID-19 disease, the infected individuals may encounter some respiratory problems occurring orderly through this phases: cellular invasion and viral replication in the nasal cavity, replication in lung and immune system activation, pneumonia, ARDS, cytokine storm, and multi-organ failure [27–29]. Many interactive factors contribute to lung tissue damage and impaired respiratory muscles in both COVID-19 and MS diseases included activated immune system and its pro-inflammatory cytokines such as IFN-ϒ, TNF-α, and IL-1β [8–10, 14, 30–35], central demyelinated lesions/plaques formed in areas monitoring respiratory rhythm, and muscles induced by the function of the activated immune system [36–46], local, and systemic (soluble) imbalance in the RAS axis [12, 13, 47, 48] (Fig. 1).

Respiratory epithelium, especially ciliary airway epithelium, is the critical point of SARS-CoV-2 entering into the host since it expresses the highest levels of SARS-CoV-2 receptors, namely the angiotensin-converting enzyme 2 (ACE2) [49–51]. Epithelium serves as a barrier against pathogens and particles, preventing tissue damage through secreting mucosa and also mucociliar clearance [24]. Upon cell–virus crosstalk and consequent entering into ciliary nasal cells, SARS-CoV-2 travels to lower respiratory tracts (LRTs) and then triggers the extreme production of inflammatory cytokines and chemokines, such as IL-1, IL-6, IL-8, TNF-α, and -β, and monocyte chemoattractant protein 1 (MCP-1). These inflammatory mediators recruit leukocytes to the infectious site [52–54]. Increased cytokine levels can devastate airways and alveolar epithelium by triggering the cells apoptotic process and formation of reactive oxygen species (ROS) exacerbating the pneumonia severity. Alveolar damage remarkably impairs gas exchange and leads to respiratory failure [24, 55, 56]. In more detail, for example, TNF-α has an important role in regulating neutrophils influx following lung damage [23, 57]. Neutrophils release toxic oxygen metabolites such as superoxide anion, hydroxyl radicals, and hydrogen peroxide which cause cellular oxidative damage in pulmonary endothelium, parenchymal cells, and inflammatory edema [58–60].

Infiltrated neutrophils, therefore, secret neutrophil extracellular traps (NETs) to control lung infection, but their high production is associated with lung damage by turning the alveolar macrophages into the pro-inflammatory M1 phenotype [61]. The main mechanisms for such transformative phenotype ascribed to the NETs induced activation of signaling pathways in pulmonary cells include extracellular signal-regulated kinases 1/2 (ERK1/2), c-Jun N-terminal kinase (JNK), p38, and nuclear factor-kappa B(NF-κB) proteins [62]. Besides, the proteins and dsDNA components located in NETs may act as critical autoantigen sources to trigger local inflammatory cascades [63]. Of note, infectious and damaged epithelium attracting the pro-inflammatory cytokines is associated with reduced secretion of surfactant proteins of A and B [64], resulting in alveolar collapse. It is important to note that COVID-19 often initiates with symptoms akin to influenza [27]. Coronaviruses are the second reason for induced common cold [65]. As mentioned, SARS-CoV-2 can worsen the conditions of some patients with asthma, since it causes infection of the upper and lower respiratory tracts [65–67]. Epithelium of upper respiratory tract needs 3 weeks to return to the previous normal level [68].

Endothelial dysfunction is another lung pathophysiology of COVID-19 disease. In these patients, extended inflammatory cytokine levels will induce some changes or damages in smooth muscle cells of lung vessels including phenotypic switching from the quiescent contractile phenotype to a proliferative, migratory, and synthetic phenotype which is associated with vessel thickening and also reticular small vessels [69, 70]. It has been reportedly illustrated that endothelial cells suffer apoptosis [71]. Increased permeability of lung vessels is another problem that patients with COVID-19 encounter, which is corroborated by alveolar hemorrhage and fibrin deposition [24]. Thus, these disorders in lung microvessels can impair vascular perfusion [71, 72]. Additionally, regarding the expression of ACE2 on microvascular endothelial cells and vascular smooth muscles, SARS-CoV-2 disrupts the relationship between endothelium and smooth muscles which results in disordered vasodilation and vasoconstriction as well as disorders in gas exchange [73]. It has been documented that some central autoimmune diseases are susceptible to other diseases involving immune system [74, 75]. MS is a chronic central disease characterized by inflammatory demyelination. In both patients with MS and its animal model, experimental autoimmune encephalomyelitis (EAE) is initiated with reactivation of T cells crossing the blood–brain barrier (BBB) into the CNS [76]. Patients with MS have a reduction in clearing virus from their lungs that in part stem from lower efficiency of their anti-viral immune responses [74]. MS patients during contracting respiratory viral infection such as influenza and pneumonia experience higher morbidity and severity than individuals without MS disease [77–80]. Thus, MS disease can exacerbate the expansion of respiratory infection that may partly refer to the regulation of inflammatory characteristics of T cells in MS patient’s lungs [43]. Interestingly, it has also been revealed that lung is involved in myelin-reactive T cells becoming pathogenic [43]. As a natural procedure, the mobilization of innate (e.g., natural killer; NK) and acquired immune (CD8 ^+^T cells) system is cardinal strategy to control the viral replacing and to clear efficiently the respiratory viruses through releasing an anti-viral pro-inflammatory cytokines, like interferon (IFN)-γ [81, 82]. A report documented that EAE animals with respiratory infection lowers the production of effector cells, both innate and acquired, and IFN-γ, suggesting a reduction in the immune response to infection in patients with MS [82]. MS and animal model of MS are also associated with mobilizing the extensive population of myeloid-derived suppressor cells (MDSCs), especially their CD11b^+^ subunit, from bone marrow, blood, spleen, and CNS into the lungs. These myeloid cells inhibit the proliferation of CD8 ^+^T cells and consequently their IFN-γ production in lungs [74, 78, 83]. MDSCs use various mechanisms to mitigate the immune response including production of IL-10 and synthesis of nitric oxide (NO) through inducible nitric oxide synthase (iNOS) [83–85]. Therefore, MS patients infected with respiratory viruses present increased viral titers, lung pathology, and consequent increases in their mortality. If the patients with MS survived from respiratory infection, their hospitalization lasted 2 times more than individuals without MS and only infected with respiratory viruses, since patients with MS are exposed to extension of relapses after infection [86–89]. It is also reported that the susceptibility of MS patients to respiratory infections may be elevated during relapsing–remitting MS. It has been suggested that patients with MS during the remission phase show a reduction in their innate immune cells. Of these cells, granulocytes (neutrophil, eosinophil, basophil) are the most important to fight against viral infections [90–93]. As a part of immune response, granulocytes migrate to the infectious site, in this case the lungs, and consequently secret effector molecules, such as histamine, cytokines, chemokines, enzymes, and growth factors [94, 95]. It has been shown that the number of granulocytes, especially neutrophils, is lower during the remitting phase and that may lead to diminished IFN-γ production, a stimulating factor of neutrophils or granulocytes, by Th1 cells [90, 96]. The other pathway that may promote the susceptibility of patients with MS to infectious diseases is immunosenescence, which is associated with progressively diminished number of naïve T cells, originated from structurally and functionally thymic involution [97, 98]. The age range of 20 to 40 is a benchmark age range where the majority of individuals may be afflicted with MS disease and live with this disease for a long time even until death [99]. The events that take place in the immunosenescence process result in poorer immune responses in the old patients with MS [100]. Thymus is a lymphoid organ, where the T cells mature, and is a main source for circulating T cells. The thymic size is progressively elevated until puberty and then undergone involution with its parenchymal tissue replaced by fat [98, 101]. Respiratory viruses are leading causes of acute respiratory infections every year affecting mainly older patients with MS and the elderly. Up to date, several reports have described the association between respiratory viral infections with neurological symptoms [102]. Thus, in MS patients, respiratory viruses have placed themselves as relevant agents responsible for CNS pathologies. Aged MS patients who are in advanced phase of the disease do not have enough CD8^+^ T cells in their circulation and consequently in their lung tissue to fight against viral antigens and increased infectious risk [98] exacerbating the neurological signs of the patients [103–106]. Lungs are inflamed during respiratory infection, which is associated with increased upregulation of a chemokine, namely CCL20, to attract Th17 cells into the lungs. Through increased gene expression encoding chemokine receptors and integrin receptors on T cells, these immune cells which converted to the pathogenic phenotype are licensed to enter circulation [43, 107]. Circulating pathogenic T cells then increase BBB permeability and lesion load and volume in brain and spinal cord [108], which is equal to worsening the clinical signs of MS patients.

Reduced physical activity during lockdowns, and especially hospitalization, causes respiratory muscle wasting and impaired skeletal muscles that could lead to sarcopenia and cachexia [109–112]. Using mechanical ventilation for several weeks is another factor involved in structural and functional impairment of respiratory muscles [111, 113, 114]. Also, diaphragm, a key inspiratory muscle, during mechanical ventilation is put in an unloaded condition which can be accompanied with atrophy and consequently weakness. Brainstem centers monitoring respiratory rhythm have been documented to switch off sending efferent impulses to respiratory muscles amid long-term usage of mechanical ventilation [115–117]. In support of this claim, reports disclosed that COVID-19 patients during their stay in intensive care unit (ICU) wards experienced diaphragm impairment and a decrease in its thickness [116, 118]. Also, an atrophy in diaphragm fibers and a reduction in its contractile function have also been reported [119, 120]. MS patients also experience such inactivity which is highly similar to those who are bedridden [121]. Inactivity-induced influence on respiratory muscles may also be ascribed to production of ROS by the pro-inflammatory cytokine storm and activated macrophages and monocytes. Reactive oxygen species and resultant oxidative stress increase the apoptosis and proteolytic processes through the expression of caspase-3 and the activation of the ubiquitin–proteasome system [119, 120, 122–127]. The ubiquitin–proteasome system is activated by hyperinflammation conditions as observed in both COVID-19 and MS diseases [128–130]. The ubiquitin system, which is dependent on ATP, is the main mechanism responsible for muscle atrophy [131, 132]. Pro-inflammatory cytokines induce muscle atrophy, particularly in respiratory muscles, through the following additional mechanisms: inhibited protein synthesis due to the changes in anabolic hormones such as insulin-like growth factor 1 (IGF-1), the mitigating function of satellite cells, attenuated expression of myoblast determining protein 1 (MyoD), downregulation of myosin heavy chain (MHC) of slow twitch fibers and increased degeneration and changes in fiber-type phenotype [133–138], increased activation of NF-κB which leads to the activation of the ubiquitin system [137, 139, 140], and hindered expression of the peroxisome proliferator-activated receptor (PPAR), which has a role in preventing inflammatory conditions, all contributing to a catabolic state along with muscle atrophy [141].

In addition to the immune system, intrinsic expression of ACE2 receptors in the skeletal muscle system may play an important role in SARS-CoV-2 entering into muscles and contribute to skeletal muscle morbidities [142]. Indeed, increased virus entrance into respiratory skeletal muscles is also associated with produced pro-inflammatory cytokines and as a consequence ROS formation. Reactive oxygen species induce muscle damage and atrophy that will finally lead to muscle fatigue [110, 143–147]. These species, further, reduce muscle force production by several mechanisms, including attenuating sensitivity of myofibrils to calcium (Ca^2+^) [148, 149], oxidizing regulatory proteins of sarcoplasmic reticulum (SR) Ca^2+^ release channels [150, 151], opening ryanodine-sensitive Ca^2+^ release channel resulting in increased Ca^2+^ concentration [150, 152], inhibiting the function of sarcoplasmic reticulum calcium ATPase (SERCA) which is necessary for ATP hydrolysis [153], impacting on myofibril structure and function [154, 155], altering cross-bridge kinetics [149], oxidizing myosin heavy chain and also increased impairment of myosin function [154, 156], and modifying the function of troponin C [157]. Thus, increment in ROS can incur in Ca^2+^ dysregulation in cell cytosol (increased intracellular Ca^2+^ concentration) that in turn activates calpain [145, 158]. Calpain causes the releasing of sarcomere proteins via cleaving cytoskeletal proteins such as titin and nebulin which are anchored to the contractile components [159]. In this context, however, future studies should address whether the direct attack of SARS-CoV-2 on respiratory muscles has a role in their atrophy.

Severe active respiratory syndrome coronavirus 2 also associates with respiratory challenges after entering the body via respiratory or neuronal pathways. Coronavirus is categorized as a virus that after entering to CNS causes lesions in brainstem, a sensitive area for respiratory cycles [3, 160]. It may be concluded that produced lesions cause a neuromuscular impairment of respiratory muscles. Demyelinated lesions which are observed in patients with COVID-19 and MS diseases are actuated by cytokine storm [18, 33, 36, 37, 161, 162]. Upon entering into the body, SARS-CoV-2 identified as a foreign antigen by immune cells triggers serious immune and inflammatory responses which as a consequence cause extensive peripheral and central release of pro-inflammatory cytokines. There is a positive correlation between increased pro-inflammatory cytokines and disease progression [163, 164]. This process suggests the lack of immune regulation in response to respiratory infection.

That the respiratory system in MS patients can be impaired has been neglected by clinicians and scientists due to prominent other signs in these patients. Altered respiratory function and respiratory muscles strength are changes exacerbated with increasing MS disabilities [165–167], and it has even been disclosed that these respiratory issues account for roughly 47% of total deaths in MS patients [168]. There are acute and chronic respiratory failures in MS patients. Respiratory failure happens in the terminal stages of MS and is usually associated with significant bulbar or limb paralysis [169]. Respiratory failure may be acute, typically secondary to demyelinating lesions in the cervical cord or the medulla, or chronic, typically found in the terminal stages of the disease and related to weak respiratory muscles, and ineffective cough, leading to aspiration, atelectasis and pneumonia. Of the two kinds, only acute respiratory failure is potentially reversible with treatment [169–172]. Weakened respiratory muscles, especially expiratory ones, are a prevalent detriment in advanced phase of MS disease [167, 173, 174]. Paraplegic progression from distal to proximal in MS causes impairment in expiratory muscles prior to the diaphragm and intercostal muscles [175]. The regulation of respiratory muscle function is controlled in the regions of the brain stem and spinal cord, dorsal, and ventral respiratory centers. MS patients have centrally demyelinating plaques extended to these respiratory centers which associate with disrupted impulses and neural pathways related to respiratory muscles [166, 167, 169, 172]. Additionally, the majority of MS patients experience autonomic dysfunction, including in the thermal system, which is originated from lesions in brain stem and medulla areas of the brain [176]. Hyperthermia induced by these lesions negatively influences impulse conduction throughout neurons present in respiratory centers that control respiratory muscles [177, 178]. The primary mechanism that can mechanistically explain such reduction in impulse conduction is attributed to the potassium channels expressed in these neurons. Hyperthermia activates two-pore domain K^+^ (K2P) channels on respiratory muscles controlling neurons and therefore culminates in neuronal hyperpolarization and reduced action potential propagation [179–181].

The above-mentioned pathways can diminish strength and endurance of respiratory muscles [167, 172–174], more predominant in expiratory respiratory ones [172, 174, 182]. The reduction in these muscle fitness components associate with changes in lung volume and capacity [172], including VC, maximal expiratory and inspiratory pressures, forced expiratory volume in the first second (FEV_1_: the volume of air exhaled in the first second during forced exhalation after maximal inspiration), FVC, FEV_1_/FVC ratio, peak expiratory flow (PEF: the highest forced expiratory flow), and total lung capacity [165, 172, 174]. Collectively, these pulmonary issues in patients with MS engender some abnormalities such as disruption in diffusion capacity of gas dispersed across alveolar membrane, ventilation to perfusion ratio, increased physiological dead space, and consequently diminished oxygenation, inefficient cough, reduced respiratory control, dyspnea, and exercise intolerance or reduced exercise capacity [171, 183–185]. All complications related to respiratory muscle impairment can put MS patients in a severe condition or even death upon infection with COVID-19.

Multiple sclerosis is always associated with some disabilities, including fatigue, strength, coordination, and cognitive signs loss, that progress over time and lead to physical and social inactivity [185]. Besides, several years ago, it has been recommended that MS patients should not participate in physical exercise, just because of increasing their internal temperature during exercise would compromise their clinical signs [186, 187]. Thus, MS patients face a sedentary live [188–190] accompanied with increasing body mass index (BMI) and obesity. Increased BMI and obesity in turn compromise MS severity and even elevate the odds on afflicting MS disease in younger ages [191–195]. Adopted sedentary lifestyle in these patients results in an imbalance between energy intake and expenditure, leading to obesity. Obesity, which is defined as a BMI ≥ 30 kg m^−2^, is a metabolic disorder with accumulating fat mass in various body points. Extra burden of body fat through mechanical limitations and reduced thoracic compliance may change lung function/physiology and respiratory rhythm [196, 197]. Hence, increased fat accumulation in areas around ribs, diaphragm, and abdominal cavity implements a mechanical load on chest cavity that abates respiratory compliance (increased stiffness) [198–200]. Elevated intra-abdominal and pleural pressures due to upward and outward movements, respectively, in diaphragm and chest wall preclude airflows toward negative pressure gradient in lungs and pleural space with the lower part of lung system tending to collapse [199, 201, 202]. Generally, increased mechanical load and internal pressures cause a change in respiratory pattern to the quick, shallow type (increased breath rate) [203]. Compromised lung volumes are secondary to the changes of respiratory pattern. The most detrimental alterations in lung volumes and capacities have been observed in expiratory reserve volume (ERV), FVC, forced residual capacity (FRC), total lung capacity (TLC), and tidal volume [204–210]. An impaired lung gas exchange, hypoventilation, and eventually hypoxia have been pinpointed in obese individuals that mostly resulted from regional ventilation–perfusion mismatching; on the other hand, the lower parts of their lungs are often under-ventilated and contrarily over-perfused [211, 212]. Adiposity is characterized by deposition of fat in adipocytes, followed by adipocyte hypertrophy and hyperplasia. The hypertrophied adipocyte are infiltrated by macrophages and they in turn release pro-inflammatory cytokines (TNF-α, IL-1β, IL-6) and adipocytokines from the TGF-β family, especially TGF-β1 [213–218]. Additionally, an imbalance between some other adipokines, including adiponectin and leptin, also occurs. The concentration of adiponectin, as an anti-inflammatory adipocytokine, and leptin, as a pro-inflammatory cytokine, respectively, decreased and increased in obese individuals [219, 220]. In a more general term, increased secretion of these adipokines into circulation can influence other organs throughout the body and produce some lung disorders, like asthma, COPD, and fibrosis [221, 222]. Increased compensatory lung perfusion in obese individuals can guide circulating TGF-β1 to lung tissue. In lungs, TGF-β1 recruits immune cells, such as eosinophils, neutrophils, macrophages, mast cells, and fibroblasts, as well as increases the production and expression of IL-8, cyclooxygenase-2 (COX-2) and prostaglandin E2 (PGE2) in airway smooth muscle cells, leading to airway inflammation and finally asthma [223–227]. Independent of inflammatory responses in airways, TGF-β1 can cause airway remodeling or fibrosis [228]. TGF-β1 modulates the synthetic and secretory functions of epithelial, airway smooth and monocyte cells, and fibroblasts. Increased function of these cells are associated with synthesis and deposition of extracellular matrix (ECM) components. These ECM components include collagen I and IV, elastin, fibronectin, and biglycan [229–231]. Adiposity as a secondary outcome to changing lifestyle in MS patients with detrimental effects on respiratory system can be a risk factor for infectious diseases such as COVID-19 [232] and therefore expose MS patients to higher mortality rate when infected to COVID-19 than non-obese ones. More importantly, accumulated adipose tissue extensively expresses ACE2 enzyme, the receptor for SARS-CoV-2 entering into cells; this tissue thus acting as a reservoir for virus [233, 234].

The infection of SARS-CoV-2 can be fatal and its severity is heterogeneous among individuals that have or do not have underlying diseases [235]. Such heterogeneity in disease severity may be attributed to the underlying diseases that already naturally promote respiratory problems or to differences in ACE2 expression and distribution [236]. Angiotensin-converting enzyme 2 presence in lung tissue can underline the promoted respiratory issues and their severity [237]. Angiotensin-converting enzyme 2 is a dipeptidyl carboxypeptidase expressed remarkably on numerous tissues and organs, including lungs, vascular endothelia, cardiovascular tissue, stomach, small intestine, colon, skin, Ranvier nodes, thymus, bone marrow, spleen, liver, kidneys, and brain [73, 162, 238, 239]. However, ACE belongs to RAS. RAS comprises two arms or axes in which one of them is detrimental/pathological and another is protective with opposing effects [240, 241]. The main pathological axes are angiotensin II (Ang II also known as Ang1-8)/ACE/angiotensin type 1 receptor (AT1R). Angiotensin-converting enzyme or ACE cleaves angiotensinogen I (Ang I) to form Ang II exerting its actions by binding to AT1R. The protective axis consists of angiotensin 1–7 (Ang1-7)/ACE2/Mas receptor (MasR), and sometimes angiotensin type 2 receptor (AT2R) is also taken into account in this axis. Angiotensin-converting enzyme 2 produces Ang1-7 via catalyzing Ang II [240, 242, 243]. The second axis has anti-inflammatory, anti-proliferative, anti-fibrotic, anti-apoptotic, and vasodilatory functions [244]. There are two RAS types, namely, systemic and local RAS [245]. Indeed, ACE2 is found in two forms, membrane associated and soluble which is catabolically activated [246, 247]. The upregulation of the detrimental axis of RAS has been observed in disease circumstances [248–250], and it has been found that the activity of soluble ACE2 decreases in disease conditions [250]. Soluble and membrane-associated ACE2 as protective axis of RAS were downregulated following infection with SARS-CoV-2 which may contribute to increased viral entering and lysing of ACE2-positive cells [12, 48, 251, 252]. On the other hand, attenuated ACE2 is associated with loss of protective effects of ACE2 and increased Ang II in both mRNA and protein levels [247]. The circulating and tissue levels of ACE2 in some diseases, such as cardiovascular and chronic kidney diseases, and also smokers with chronic obstructive pulmonary disease (COPD) are increased as a compensatory response and it may be an explanation for why some persons with underling diseases are at higher risk of COVID-19-induced mortality [253, 254]. Increased plasma level of Ang II has been also reported in patients infected with SARS-CoV-2 which had positive correlation to viral load and lung damage. Therefore, it may be possible to inhibit the detrimental effects of COVID-19 through suppressing of Ang II [12, 252, 255, 256]. Angiotensin II or Ang II exerts its pro-inflammatory and pro-fibrotic action through binding AT1R on lung cells [247]. Contrarily, the protective role of the ACE2/Ang1-7/MasR axis has been identified in several models of lung damage including initial type of SARS [252]. Angiotensin-converting enzyme 2 suppresses the production of Ang II, the activity of ACE and AT1R activation in order to prevent severe lung failure by mediating the production of bioactive peptide of Ang1-7 which activates MasR and AT2R signaling [257–260]. Angiotensin 1–7 promotes its beneficial functions by inhibiting ERK1/2 and natural NF-κB pathways and also prevents bronchial responsiveness, which is a hallmark characteristic of chronic asthma [258, 259]. In MS patients associated with SARS-CoV-2, notable differences were observed in the numbers of lung NK cells, CD8^+^ T cells, inflammatory monocytes, and myeloid-derived suppressor cells (MDSCs). This leads to increased lung cell infiltration, suppressive monocytes in the bone marrow, blood, spleen, and CNS, and a decrease in anti-viral CD8^+^ T-cell function. It is worth noting that increased concentration of ACE, and dysregulation in ACE/ACE2 balance, has been observed in diseases associated with ARDS, like in patients with severe COVID-19. Produced imbalance favors the detrimental axis of RAS, which can impair lung function due to inflammation, fibrosis, and lung edema, the latter resulting from promoted permeability of lung blood vessels [252, 258, 261, 262].

Majority of the human studies have measured soluble ACE2 in blood, while membrane-associated ACE2 assessment needs more investigation in future. It has been acknowledged that using ACE2 blockers or antibodies disrupting viral entering into the cell during COVID-19 infection, may endanger patients, since these strategies abate the protective effects of ACE2 and its anti-inflammatory activity and as a consequence promote lung susceptibility to damage [263, 264]. Furthermore, utilizing analogue receptors or recombinant soluble ACE2 is another strategy to reduce viral binding in a competitive manner to membrane-associated ACE2 and finally through this procedure mitigate infection and viral load. In this context, soluble ACE2 acts as a decoy receptor and reduces the binding of SARS-CoV-2 to local/membrane-associated ACE2 and as a result reduces lung damages induced by COVID-19 disease [264, 265]. Of note, based on evidence, increased levels of soluble ACE2 point to the attenuation of membrane-associated ACE2 levels [266].

As mentioned above, COVID-19 patients have a lower protective axis compared with controls or rather, patients with COVID-19 disease illustrated higher circulatory Ang II levels which were correlated to viral load [255, 267].

Angiotensin II receptor blockers (ARBs) improve ACE2/Ang1-7/MasR axis of RAS which is associated with assuaged ROS production, inhibiting lung fibrosis via mitigation of collagen deposition, reducing the disruption of alveolar walls through anti-inflammatory influences mediated by suppression of NF-κB pathway, and also by reducing the production of pro-inflammatory cytokines (IL-6, TNF-α) [268–271]. Collectively, RAS manipulation may abate SARS-induced tissue damages [12].

In any case, details about the expression and distribution of ACE2 receptors are scarce in MS patients and they should be identified in future research. SARS-CoV-2’s receptor availability can increase the virus entering into the lung cells and worsen the disease complications [236]. Both ACE2 and transmembrane serine protease 2 (TMPRSS2) are expressed on the apical membrane of alveolar cell type 2 (AT2). The virus binds to the ACE2 receptor through its spike glycoprotein (S) and then TMPRSS2 helps SARS-CoV-2 to fuse with the host cell membrane for the release of its genome [272]. Thus, other factors such as TMPRSS2 may be critical in regulating COVID-19 disease, although it remains to be clarified in future research. As above mentioned, RAS has been found peripherally in circulation and centrally in the CNS [273]. The main sources of RAS components in CNS are glial cells (especially astrocytes) and neurons [274]. Increased expression and activation of detrimental components of RAS have been reported in circulation, cerebrospinal fluid (CSF) and brain tissue (especially on lesions) of MS patients [13, 47, 275], while there was a reduction in the protective ACE2 component [13]. This observed status in MS patients is associated with exacerbating neurological signs [259, 276, 277]. Importantly, the detrimental axis is activated in the early steps of experimental autoimmune encephalomyelitis (EAE), as an animal model of MS, but the protective axis is activated during the end time point of this model [278]. The majority of studies have concentrated on the inflammatory role of the detrimental axis of RAS. Growing scientific literature, using the EAE model, has reported that ACE inhibitors and ARBs and improvement of the protective axis can attenuate the clinical scores and inflammation [243, 279–281]. Thus, RAS axes should be taken into account for therapeutic purposes in the treatment COVID-19.

A correlation has been detected between pulmonary damage and changes in its function [282]. Recovered patients from COVID-19 still experience impairments in pulmonary functional capacity for several months [283]. Such functional disorders or functional reduction have been proved in forced expiratory flow, forced expiratory volume in 1 s to forced vital capacity (FEV1/FVC) [284]. Otherwise, ground-glass opacities are observed in the early and progressive phases of the disease [285]. Patients’ age, comorbidities, history of cigarette smoking, the duration of hospital admission, and also the type of medication administration are the critical determinants in the severity of pulmonary disorders [283, 286].

It has been shown that there is a mutual relationship between having chronic respiratory disease and increasing cerebral infraction; on the other hand, it is shown that there is a significant relationship between impaired respiratory function and both brain atrophy and volume of white matter lesions [5, 167, 171, 287].

Remarkable brain and brainstem demyelination influence motor pathways, especially those that innervate limbs, which lead to mobility weakness or impairment. Multiple sclerosis is a neuro-inflammatory and demyelinated disease associated with lesions throughout the CNS, which depending on the involved brain area incurs in some disabilities [288, 289]. Pulmonary dysfunction manifested in MS primarily include impaired respiratory muscles that result in pulmonary weakness and cough. Expiratory muscles are probably more at risk to suffer impairment. It must be mentioned that there is a close correlation between disease severity and higher reductions in respiratory muscles force. In this context, after pulmonary function tests (PFTs) it has been indicated that MS patients have a low vital capacity (14%) in the supine position. Thus, respiratory dysfunction in MS patients may partly reflect the demyelinated lesions in the monitoring area of respiratory centers in the brainstem and cervical spinal cord and as a result, they can weaken the expiratory respiratory muscles. As a whole, impaired expiratory respiratory muscles may be accompanied by a higher risk of respiratory infections like pneumonia. Respiratory infection-induced mortality of MS patients is twice times higher than the general population, [172, 173, 287, 290–293]. It is worth noting that the side effects of some MS-modifying drugs such as Fingolimod, Tranquilizes, muscle relaxants, and opioids may be the main factor in the reduction of some lung function values and slowdown the ventilation action.

It has also been shown that pneumonia is the common consequence in all coronavirus and MS patients. Respiratory problems in MS patients initiate by disease progression. The systemic pro-inflammatory milieu in MS patients alone contributes to skeletal muscle weakness and these complications may increase with COVID-19 infection. In this context, it is necessary to identify the direct attack of SARS-CoV-2 on skeletal muscles in future research.

Additionally, both diseases share the same initial mechanisms and symptoms; thus, individuals with MS may experience and be placed in the intolerable condition after coronavirus infection, just like resurging the chronic relapses increasing the clinical symptoms that may lead to their death [294, 295].

### Pleiotropic roles of physical exercise

**Fig. 2:**
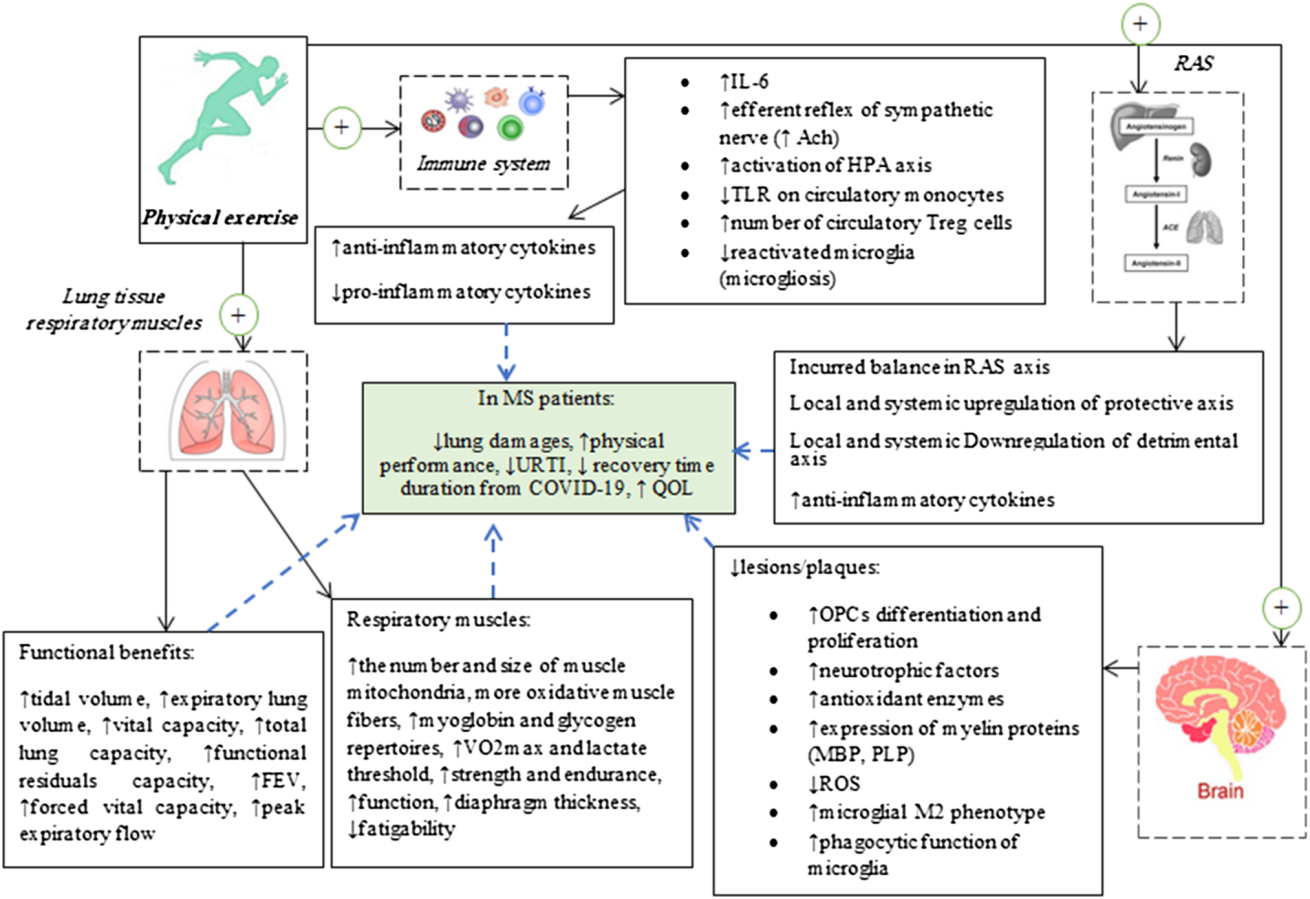
The schematic diagram of protective or modifying role of physical activity. The positive effects of regular physical exercise on four body systems is indicated by dashed rectangles and also the positive marks. The effects of positive changes in MS patients are, then, identified by the solid rectangle and also dashed arrows for every item. *FEV* forced expiratory volume; *URTI* upper respiratory tract infection; *COVID-19* coronavirus disease-19; *QOL* quality of life; Ach, acetylcholine; TLR, toll-like receptor; HPA, hypothalamus–pituitary–adrenal axis; *RAS* renin–angiotensin system; *Treg* T regulatory cells; *OPCs* oligodendrocyte precursor cells; *MBP* myelin basic protein; *PLP* myelin proteolipid protein; *ROS* reactive oxygen species

Physical exercise is a challenge on approximately the whole-body system. The movement demands, the control of skeletal muscles, the cardiovascular, and particularly the pulmonary system helps to maintain the intensity of a given exercise for longer times. Persistent contributions of regular exercise, specialty endurance mode causes adaptations in all of these physiological systems [296, 297]. The majority of respiratory muscles including expiratory and inspiratory muscles are skeletal muscles [298]. In admitted COVID-19 patients with a severe condition of mechanical ventilation, it is necessary to strengthen pulmonary muscles during the recovery period [24]. Furthermore, individuals with changes in the motor system and generally with disabilities, most probably experience functionally respiratory disorders [299]. Endurance training might be one recovery strategy to improve the function of pulmonary muscles (Fig. 2). It has been revealed that endurance training primarily increases the number and size of mitochondria and capillaries in skeletal muscles and as a consequence converts the fibers phenotype to the more oxidative type [296, 297, 300–302]. Increased myoglobin and glycogen content and the increase use of fat as a fuel source are other adaptations that occur in skeletal muscles [303, 304]. The main functional alterations in the respiratory system induced by endurance training are as following: (1) increased tidal volume and breath rate which collectively promote maximal pulmonary ventilation and (2) improved pulmonary perfusion as a result of increased pulmonary blood flow in the higher area of the lungs [305]. These adaptations in pulmonary muscles and in respiratory function are partly amenable to increased VO_2max_ and lactate thresholds. A study conducted on severe acute respiratory syndrome (SARS) survivals showed that 6 weeks of combined training (endurance and resistance) improved cardiopulmonary and muscle (upper and lower limbs) fitness and performance, increased predicted VO_2max_, and elevated health-related quality of life (QOL) [306]. Increased VO_2max_/VO_2peak_ induced by exercise training mainly comes from improving and reducing blood circulation and pressure, respectively, as well as refining cardiovascular function [307]. Additionally, a reduction in breathlessness and an improvement in muscle endurance and strength can increase contribution to physical exercise and independency in doing personal duties, all promoting QOL [308–310]. Thus, MS patients with the contribution of progressive endurance training following SARS-CoV-2 can expedite their recovery and also improve their quality of life through independence from others in daily tasks [305].

Nowadays, the training of respiratory muscles is the newest training trend to rehabilitate individuals who have a problem with their respiratory muscles or even to enhance performance in persons whose professions benefit from improving the strength of respiratory muscles [311, 312]. This training model is implemented in guise of trained expiratory and inspiratory respiratory muscles, and a combination of them [313]. It has been proved that this type of rehabilitating training in MS patients is involving positive adaptations and improvements in respiratory muscle strength, spirometer parameters, cough efficiency, fatigue, and dyspnea [290, 314–317]. Besides, respiratory training improves the strength and endurance components of respiratory muscles and as a result promote lung functional capacity and performance [318]. Due to enhancement of components related to respiration, such as slowdown breathing rate and assuaged carbon dioxide production, dyspnea is diminished secondary to the respiratory training [317, 319]. Inspiratory muscle training has affirmative effects on cardiac function by involving in autonomic nervous system; for example, increasing parasympathetic activity [320, 321]. Elevated exercise-mediated intrathoracic pressure triggers baroreflex activity leading to promoted venous return which in turn mitigates heart sympathetic activation during resting condition [175]. Despite potential influences on respiratory muscles, exercise training defies the cardiac problems incurred in MS and COVID-19 diseases and therefore prevents exacerbating ventilation process. There are several training models escalating respiratory muscle strength and endurance [322–324]. One of these models is swim training [299].

Swim training increases respiratory work; hence, this training type promotes pulmonary volumes by strengthening the respiratory muscles, especially the diaphragm [325].

Other functional changes in the form of adaptations that occurred in the pulmonary system induced by exercise training include (1) reduced fatigability, (2) increased expiratory lung volume, (3) elevated vital capacity, (4) increased diaphragm thickness, (5) enhanced function of inspiratory muscles [326, 327], (6) increased TLC, (7) promoted FRC [299], (8) increased FEV1 [299], (9) promoted FVC, (10) increased PEF [299, 328, 329], and (11) increased strength and endurance of respiratory muscles [330].

Maintenance of diaphragm activity under mechanical ventilation may prevent its atrophy [331]. Otherwise, it has been identified that increased concentration of metabolites in respiratory muscles may partly explain the fatigue of exercising organs; in such a way, metabolites trigger the firing rate of afferent nerves to the autonomous nervous system.

Increased strength of outflow of sympathetic nerve, by corollary, causes vasoconstriction and as result fatigue in exercising organs [332, 333]. Inspiratory and expiratory muscle training inflict a load on the diaphragm and as a result, increases cross-sectional area and strength and endurance of the diaphragm and also improves fatigue tolerance [334–336].

Single exercise sessions, or acute exercise, impact on the immune system by recruiting leukocytes from other organs to circulation, acquiring active phenotype of both innate and adaptive cells including NK cells, active T and B lymphocytes [337, 338], and increased release of immune modulatory peptides, such as anti-inflammatory cytokines [339]. Thus, acute exercise causes the immune activation and this may influence defense mechanisms against pathogens. Although the increased immune function may be efficacious in healthy persons, this condition can aggravate the circumstance of MS individuals particularly those who suffer from COVID-19. It is documented that regular physical exercise can attenuate respiratory issues through effectuating positive responses of the immune system or reducing pro-inflammatory cytokines as causative agents of respiratory issues in COVID-19 and MS patients (Fig. 2) [29, 340, 341]. IL-6 may be one of the outstanding mechanisms by which exercise induces a mitigated inflammatory environment. Exercise training increases the production of IL-6 from adipocytes, macrophages, monocytes, brain, liver, and skeletal muscles [340, 342, 343]. The increased circulatory concentration of IL-6 is associated with attenuated production of pro-inflammatory cytokines (TNF-α, IL-1β) from inflammatory cells [340, 342] as well as promoted anti-inflammatory cytokines, such as IL-1 receptor antagonist (IL-1ra), IL-4, and IL-10 [342, 344]. Furthermore, blockage of IL-1β receptors, which inhibits its signal transduction, maybe another anti-inflammatory function induced by IL-6 [345]. Produced anti-inflammatory cytokines reduce antigen presentation by antigen-presenting cells (APCs) which are necessary to maintain inflammatory responses [346]. The upregulation of IL-6 in lung tissue after exercise training has also been shown in lung injury in animal models [347]. IL-6 dampens pulmonary inflammation through increasing superoxide dismutase (SOD) and also restricts the disruption of alveolar barrier induced by neutrophils [348, 349]. A negative correlation between IL-6 and IL-10 has been shown with neutrophils density in lung tissue. Increased concentration of IL-6 and also activation of the hypothalamus–pituitary–axis (HPA) induced by physical exercise increase the release of cortisol, a circulatory anti-inflammatory factor. Initially increased exercise-induced cortisol reduces pro-inflammatory production by acting on its own receptors on immune cells [340, 344]. It has also been shown that IL-6 can activate HPA per se [348, 350, 351]. The previous evidence corroborates this claim since an increase in IL-6 receptors and an enlargement have been observed in adrenal glands [350]. Exercise directly mitigates pulmonary inflammation by increasing glucocorticoid receptors on inflammatory lung cells. It also dampens the levels of pro-inflammatory cytokines in inflammatory lung tissue induced by endotoxin in animal models [347, 348]. Reduced pro-inflammation plays a critical role in abating the permeability of microvascular endothelium [352] and accordingly reduces ROS and lung edema [62]. Importantly, it has been revealed that enhanced pulmonary antioxidants, particularly SOD, induced by regular exercise can attenuate ARDS produced through viral infection. Enzymatic antioxidants degrade free radicals culminating in the reduction of lung damages [62, 353–355].

Exercise training has an extensive effect on the vague tone of the parasympathetic nerve. The increased efferent reflex of a sympathetic nerve is associated with releasing acetylcholine (Ach) from its terminals. Ach binds to nicotinic receptors on immune cells attenuating the production of pro-inflammatory cytokines as well as acts on macrophages by converting their phenotype from M1 (pro-inflammatory phenotype) to M2 (anti-inflammatory phenotype) [356, 357]. Reduced toll-like receptors (TLRs), especially TLR4, on circulatory monocytes may be another way through which exercise impacts the changes of immune status. Activated intracellular signals of these receptors trigger the production of pro-inflammatory cytokines [358, 359]. Therefore, regular exercise revolves negative immune response to a positive one. Another change in immune function resulting from exercise training is increased circulatory number of T regulatory (Treg) cells [360]. These cells secret anti-inflammatory cytokines like IL-10 and transforming growth factor-beta (TGF-β) and also increase the proportion of Th2 to Th1 which is related to promoting anti-inflammatory cytokines [361]. There is cross-reactivity between pro-inflammatory cytokines and microglial cells; in such a way, reduced pro-inflammatory cytokines induced by exercise training, mitigates reactivated microglia (microgliosis) and consequently decreased microgliosis associated with assuaging the produced pro-inflammatory cytokines released by reactive microglia [362, 363]. The changes of detrimental immune responses to reparative/positive responses may be efficacious to mitigate pulmonary damages resulting from COVID-19 disease and to improve the strength and endurance of the respiratory muscles in MS patients infected with COVID-19. Importantly, attenuated pro-inflammatory cytokines provided by physical exercise can also be beneficial for reducing neuronal loss and for reducing the demyelination induced by MS/COVID-19 in brain areas monitoring respiratory muscles and ventilation cycle [32, 364, 365]. Thus, exercise training establishes an appropriate balance in lung infection, tissue homeostasis, and immune response.

It has been found that exercise inhibits alveolar macrophages polarization to pro-inflammatory M1 phenotype by reducing NETs production and suppressing ERK1/2 and NF-κB pathways in lung cells and macrophages. This action can culminate in the mitigation of lung damages [366]. Besides, exercise enhances sputum clearance throughout the pulmonary system, which can be attributed to increased activity of nasal epithelial sodium channels (ENaC), promoted ventilation, shear force, and body movements [367]. Damped neutrophilic inflammation has been reported in individuals with pulmonary problems after participation in regular exercise programs and is associated with the diminishment of complement receptors [368]. Thus, exercise could reduce lung inflammation induced by infection, particularly in patients with MS.

Increased remyelination, or rather, ceased demyelination mediated by regular physical exercise could be attributed to the following: (1) increased central expression of neurotrophic factors and their receptors expressed in brain areas, particularly on oligodendrocyte precursor cells (OPCs), which can elevate the proliferation and differentiation of OPCs to adult (myelinating) oligodendrocytes enveloping neural axon [369, 370], (2) increased number of mitochondria, which is associated with mitigating the production of pro-inflammatory cytokines, reduces myelin damage induced by oxidative stress [371], (3) increased antioxidant enzymes [372], (4) upregulation of some myelin protein expression, such as myelin main protein (MBP) and proteolipid protein (PLP) [373], which are expressed on myelin sheath and also essential for myelin formation and thickness [374, 375], and (5) phenotypic conversion of microglia from M1 (pro-inflammatory) to M2 (anti-inflammatory) type and maintain them in inactivation or resting state as well as increasing their phagocytic function for expediting clearance of debris [376, 377]. Collectively, contribution in regular physical exercise can preclude plaque/lesion extension to the areas of the brain more related to respiratory centers and even restore nerve impulses through remyelinating processes. The relationship between immune and pulmonary systems in MS individuals with COVID-19 disease is per se complex and there is no information related to exercise training and respiratory system in MS patients who have been infected with COVID-19.

It has been postulated that low-to-moderate-intensity exercise in contrast to high-intensity exercise, causes a decrement in upper respiratory tract infections (URTI) and symptoms [378]. Besides, the individuals with moderate exercise levels also experience lower URTI incidence compared to their sedentary counterparts [379]. The main mechanisms related to reducing URTI induced by moderate regular exercise training have been attributed to the following (Fig. 2): first, increased salivary immunoglobulin A (s-IgA) which is the first line of the body defense against foreign pathogens, like respiratory viruses. This factor binds to respiratory viruses and eliminates them through opsonization [380, 381]. Second, immune phenotype changes from T helper 1 (Th1) to Th2 (improving Th1/Th2 balance). Th1 cells produce pro-inflammatory chemokines when exposed to pathogens, but their excessive responses can incur in tissue damages in the lungs [382]. In this context, moderate exercise training attenuates immune cells infiltration to lungs and lymph nodes drainage and release of pro-inflammatory cytokines by Th1 are reduced [383]. Third, increased IL-2 levels in lung tissue enhance differentiation and maturation of Treg cells. Increased number of Treg is congruent with establishing an anti-inflammatory milieu in lungs [382]. Anti-inflammatory cytokines such as IL-4 exert another role in reducing detrimental pro-inflammatory conditions. Interleukin-4 facilitates the differentiation of naïve Th to Th2 phenotype which has an anti-inflammatory function as well as co-stimulates B cells to secrete virus-neutralizing antibodies. Viral antibodies reduce the virus load through inhibiting the infection of cells and opsonizing the infected cells [383]. Fourth, increased soluble TNF-α receptor which is capable to bind to circulatory TNF-α through which mitigates membrane-binding propensity and consequently reduces activation of NF-κB signal pathways. Fifth, an increment in eosinophil chemoattractants causes extravasation of eosinophils into the infected lung tissue where their ribonucleases can degrade virus’s single-stranded RNA and suppresses virus replication [383]. Initial increases in cortisol induced by chronic exercise may act as an assuaging factor of pro-inflammatory condition produced by infection and as a consequence reduces lung susceptibility to infection [383, 384]. In this matter, professionals should be aware that prescribing a proper exercise protocol in MS patients with SARS-CoV-2 infection is essential, since higher core body temperature (hyperthermia) in individuals with MS may act as an endogenous stress factor that causes a higher CNS recruitment and higher exertion. In this case, higher exertion will lead to increased concentration of stress hormones and result in impairment of the host immune system which it may endanger MS patients with compromised immune system [385, 386]. Sixth, exercise increases the circulation of IL-6 derived from exercising skeletal muscles and, it, in turn, upregulates anti-inflammatory cytokines, including IL-1ra and IL-10. These anti-inflammatory cytokines mitigate the extended inflammation originated from respiratory virus infection [383, 387]. Besides, increased recruitment of NK and cytotoxic T cells also occurs following regular exercise training, improving immune defense against foreign pathogens [388, 389]. Exercise-mediated increases in immunosurveillance and attenuated inflammation have been observed in some parts of the body, including the upper respiratory tract (URT), lung, blood, and skeletal muscles, among others [389, 390]. Thus, regarding a reverse relationship between mediated exercise training and URTI incidence and duration [391, 392] and also fatality and pneumonia rates [393–395], either individuals with a clinical condition or healthy are encouraged to regularly practice physical exercise. It is worth noting that highly fitted persons have lower basic levels of inflammatory biomarkers compared with unfitted ones [396].

As mentioned, host susceptibility to SARS-CoV-2 is dependent on binding between host ACE2 and spike (S) glycoprotein of the virus which is known as the S1 subunit [397]. Although there are not enough reports regarding exercise training on ACE, especially ACE2 as local or lung tissue receptor, the changes of other subunits in other organ systems like kidneys, heart, brain, skeletal muscles, and circulation mediated by exercise are available [398, 399]. The most beneficial and prophylactic effects of exercise maybe induced through changes in RAS (Fig. 2) [400, 401]. Based on a literature review and recently original reports, regular exercise downregulates systemic and local ACE/AngII/AT1R axis and also upregulates all components of ACE2/Ang1-7/MasR axis, as well as transfers the axis balance to the protective axis [400, 402, 403]. Upregulated protective axis of RAS increases the bioavailability of prostaglandins (PGs) and bradykinin as well as enhances anti-inflammatory environment, augments anti-fibrotic and antioxidant defenses, and normalizes oxidative stress and anti-apoptotic environment [404–406]. These responses in RAS can improve lung blood flow and consequently lead to reduced oxygen deficiency in MS patients infected with COVID-19 [407, 408]. Besides, it has been claimed that exercise training reduces lung lesions and fibrosis through the normalization of RAS axes and reducing collagen deposition [271, 399, 409]. By affecting this system, exercise training can attenuate the susceptibility of individuals to detrimental functions of COVID-19 infection or mitigate the severity of disease by the following additional strategies: (1) mitigated severity of comorbidities [247] and as a result reduced COVID-19-induced mortality rates [410, 411] and (2) warded off the diminishing effects of COVID-19 on ACE2 via increasing ACE2 activity and its concentration [412], although the positive or negative effects of increasing ACE2 should be investigated. Thus, RAS manipulation and its normalization may be a potential treatment for health optimization against the COVID-19 pandemic.

Since adipose tissue can play a role as the viral reservoir [233] and in the sense that obesity causes many structural and functional issues in respiratory system, weight loss via lifestyle changes may reverse such respiratory problems [413]. Physical exercise has profound effects on body composition by increasing fat oxidation and improving muscle mass, which has a leading role on fat oxidation and consequent weight loss. By the same token, exercise should have enough intensity to influence lipid oxidation and metabolic factors [414]. There are several pathways by which exercise causes weight loss, including increased aerobic capacity measured by maximal oxygen consumption (VO2 max) and altered body composition resulting in part from elevating muscle mass [415–417]. Promoted muscle mass is associated with more consumption of glucose and lipid as fuels and as a result dampens insulin resistance [418, 419]. In addition, increasing activation of AMP-activated protein kinase (AMPK) and peroxisome proliferator-activated receptor gamma coactivator 1 (PGC)-α is another mechanism through which exercise facilitates lipid and glucose oxidation [420, 421]. PGC-α increases aerobic capacity of muscle tissue by impacting on mitochondrial biogenesis [422, 423]. Besides, changes in some genes involving in lipogenesis and lipolysis are another adaptation that occurs during and after exercise. In this context, it has been disclosed that lipolysis [peroxisome proliferator-activated receptor (PPAR)-α, cytochrome c oxidase (COX) IV] and lipogenesis [fatty acid synthase (FAS), and acetyl-CoA carboxylase (ACC)] genes are upregulated and downregulated, respectively [424]. The initiation of exercise elevates catecholamine hormones, including adrenaline and noradrenaline. Upon release, these hormones bind to their β-adrenergic receptors expressed on adipose tissue yielding intracellular signal and consequent phosphorylation of hormone-sensitive lipase to promote lipolysis in this tissue [425]. It is worth noting that exercise reverses increased adiponectin induced by obesity. Elevated anti-inflammatory adipokine increases the expression of farnesoid X receptor (FXR) as a regulator of multiple metabolic pathways. FXR then activates adaptor phosphotyrosine protein interacting with the PH domain and leucine zipper 1 (APPL1) to increase lipolysis [426, 427]. Additionally, physical exercise establishes a balance among some adipo-myokines, such as myostatin (MST), TGF-β1, and activin A, as members of the transforming growth factor-β superfamily (TGF-β) and follistatin (FST). These adipo-myokines, particularly TGF-β members, are upregulated in adiposity and inflammatory condition, while FST inhibits their function through binding to them. Generally, FST increases muscle mass and consequently reduces body fat [428–432]. Therefore, physical exercise is a dynamic lifestyle that mitigates weight gain or obesity, as a risk factor for severe COVID-19, in MS patients and as a result, reverses the changes in lung mechanics and function. It has been suggested that weight loss associates with improving in peak expiratory flow and some spirometer indices [433–435] markedly increases in lung volumes (TLC, FRC, ERV) [436–438], diminishing airway hyper-responsiveness in asthmatic and non-asthmatic obese individuals [434, 439, 440].

As mentioned in the previous section, increased core body temperature in MS patients can influence the respiratory center and nerves in the brain monitoring respiratory muscles and ventilation rhythm. Thus, improving heat strain engendered in MS patients during coronavirus infection, as a febrile virus, would help MS patients to reduce the detrimental effects of hyperthermia on respiratory muscles, especially their fatigability [441]. Although physical exercise is notorious as a heat stressor, long-term exposure to physical exercise is associated with some adaptations in thermal regulation to diminish its compromised effects [442]. Exercise training causes adaptive changes in the cardiovascular system and hemodynamic and hematological factors, including increased contractile strength of cardiac muscle, increased plasma volume, and reduced vasoconstriction at the subcutaneous level [443–445]. These adaptations are associated with supplying deep or core organs with higher cardiac output and followed by transferring the core temperature to the body surface [446, 447]. Exercise increases antioxidant enzymes and therefore reduces and elevates reactive oxygen species (ROS) production and nitric oxide (NO) bioavailability, respectively [448–451]. Besides, increased plasma ATP concentration in response to exercise-induced hypoxia and shear stress, interacts with P2Y receptors to elevate the vasodilation factors, such as NO and prostaglandin E2 (PGE2). These exercise-induced alterations attenuate vascular damages and promote microvessel dilation [452–460]. Some other adaptive mechanisms yielded by exercise amenable to dampening core body temperature are increased sweat rate through elevating cholinergic sensitivity, higher efficiency of eccrine sweat gland in sweat production per each gland, increased number and sensitivity of muscarinic receptors responsible for sweating [442, 461]. Therefore, exercise abates the threshold for commencing subcutaneous blood flow and sweat production in response to promoting core body temperature. Generally speaking, maintaining core body temperature in a narrative range mediated by exercise can preserve the normal impulses along neurons enervating respiratory muscles, followed by the attenuation of clinical signs and premature whole and respiratory fatigue in MS patients.

## Conclusion

Our review investigated molecular mechanisms of respiratory impairments and lung damage in MS patients with COVID-19. We found that regular exercise training changes the responses of the immune system and also increases some aspects of innate and adaptive immunity against SARS-CoV-2 virus to cope with lung damages. Generally speaking, physical exercise training can mitigate the negative effects of COVID-19 disease on lung tissue and respiratory muscles in MS patients and expedites their recovery following COVID-19 infection.

## Data Availability

Data sharing not applicable to this article as no datasets were generated or analyzed during the current study.

## Abbreviations

ACE2: Angiotensin-converting enzyme 2
Ang I: Angiotensinogen I
Ang II: Angiotensin II
ARDS: Acute respiratory distress syndrome
AT1R: Angiotensin type 1 receptor
Ca^2+^: Calcium
CNS: Central nervous system
COVID-19: Coronavirus disease-2019
MasR: Mas receptor
MS: Multiple sclerosis
NETs: Neutrophil extracellular traps
NF-κB: Nuclear factor-kappa B
NK: Natural killer
RAS: Renin–angiotensin system
ROS: Reactive oxygen species
SARS-CoV-2: Severe acute respiratory syndrome coronavirus 2
URTI: Upper respiratory tract infection
